# New Si-based multilayers for solar cell applications

**DOI:** 10.1186/1556-276X-6-156

**Published:** 2011-02-18

**Authors:** R Pratibha Nalini, Christian Dufour, Julien Cardin, Fabrice Gourbilleau

**Affiliations:** 1 CIMAP UMR CNRS/CEA/ENSICAEN/UCBN, 6 Bd. Maréchal Juin, 14050 Caen Cedex 4, France

## Abstract

In this article, we have fabricated and studied a new multilayer structure Si-SiO_2_/SiN_*x *_by reactive magnetron sputtering. The comparison between SiO_2 _and SiN_*x *_host matrices in the optical properties of the multilayers is detailed. Structural analysis was made on the multilayer structures using Fourier transform infrared spectroscopy. The effect of specific annealing treatments on the optical properties is studied and we report a higher visible luminescence with a control over the thermal budget when SiO_2 _is replaced by the SiN_*x *_matrix. The latter seems to be a potential candidate to replace the most sought SiO_2 _host matrix.

## Introduction

The third generation of solar cells aims at reducing the cost and at improving the efficiency. Thin film solar cells based on silicon nanostructures is one of the most researched system to achieve such a target [1-3]. Ever since the discovery of the visible luminescence of the porous Si by Canham [4] various research groups have exploited the room temperature photoluminescent nature of silicon by fabricating different kinds of Si-based nanostructures. The luminescence is attributed to the quantum confinement of carrier in Si-nanoclusters (Si-nc) [5-8]. Among the methods of obtaining the Si nanostructures we cite electrochemical etching [4,9], fabrication of silicon dots by plasma sputtering technique [10], and multilayer approach [8,11,12].

The important part of the ongoing research involves Si-nc embedded in an amorphous matrix such as SiO_2_, SiN_*x*_, or amorphous silicon. Though Si-nc embedded in SiO_2 _is the most common structure, the problem of carrier injection in this matrix comes as a major drawback owing to the large band gap of SiO_2_. Hence the replacement of SiO_2 _by other dielectric matrices with smaller bandgap turns out to be a solution. SiN_*x *_matrix meets up these requirements and hence Si-nc embedded in SiN_*x *_matrix has become a material of choice in the recent past. In this article, we develop a new multilayer composition silicon-rich silicon oxide (SRSO)/SiN_*x *_to overcome the insulating nature of SiO_2 _by taking advantage of the reduced bandgap in SiN_*x*_. Using SiN_*x *_as the host matrix favors the electrical conductivity of carriers while we still maintain the quantum confinement as done with the SiO_2 _matrix. This study aims at fabricating and comparing the light emission properties of three different kinds of multilayer compositions: (a) SRSO/SiO_2_, (b) SRSO/SiN_*x*_, (c) SiN_*x*_/SiO_2_. Such a study is important to understand the influence of host matrices on the Si-nc and consequently to achieve an optimized solar cell device in the future.

## Experimental details

Three kinds of multilayer structures were fabricated on 2" Si wafer by reactive magnetron sputtering comprising 50 patterns of SRSO/SiO_2_, SRSO/SiN_*x*_, and SiN_*x*_/SiO_2_. We define the gas flow rate as *r*_g _= *f*_g_/(*f*_g _+ *f*_Ar_) where *f*_g _represents the N or H_2 _gas flow and *f*_Ar _represents the Argon gas flow. The SiO_2 _sublayer was fabricated by sputtering the SiO_2 _cathode under pure Ar plasma. Reactive magnetron sputtering, an approach developed by our team, was used for the fabrication of SRSO sublayers. It takes advantage of the oxygen reducing capacity of hydrogen when introduced into the Ar plasma [8]. The hydrogen-rich plasma favors Si excess in the SiO_2 _sublayer. Besides this in order to facilitate a higher incorporation of Si in the matrix, both SiO_2 _and Si cathodes were used to fabricate the SRSO sublayer. The powers of SiO_2 _and Si were maintained as 7.4 and 2.2 W/cm^2^, respectively. The hydrogen rate *r*_H _was maintained at 50% while the total flow *f*_g _+ *f*_Ar _was fixed at 10 sccm. The pressure in the chamber was chosen as 3 mTorr. Thus the SRSO/SiO_2 _multilayer structure was deposited by an alternative reactive sputtering under hydrogen-rich plasma for the SRSO layer and pure Ar plasma for the SiO_2 _sublayer. The SiN_*x *_layer was fabricated by sputtering the Si cathode and simultaneously introducing nitrogen into the Ar plasma. The nitrogen rate *r*_N _was kept at 10% while the total flow rate was fixed at 10 sccm. The pressure in the chamber was chosen as 2 mTorr for SiN_*x *_layers. The temperature of deposition was maintained at 500°C for all the cases. The thickness of the SRSO sublayer was fixed to be 3.5 nm in order to be within the quantum confinement regime. In order to understand the influence of SiN_*x *_matrix, two different thicknesses of the SiN_x _sublayer (3.5 and 5 nm) were chosen.

The FTIR spectra of these samples were recorded in absorption configuration using Nicolet Nexus spectrometer at Brewster's angle (65°). The photoluminescence (PL) spectra of the annealed samples were obtained in the visible range using Jobin Yvon monochromator in the wavelength range 550-1100 nm. The excitation wavelength of 488 nm (Ar laser) was used for measurements.

## Results and discussions

### FTIR spectroscopy

**Figure 1:**
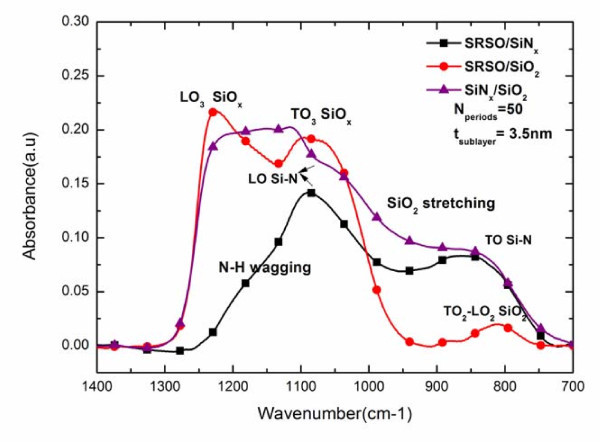
**FTIR spectra of the multilayer structures at Brewster's angle**.

Figure 1 shows the FTIR spectra obtained for the non-annealed SRSO/SiO_2_, SiN_*x*_/SiO_2_, and SRSO/SiN_*x *_multilayers. The spectra were recorded at the Brewster angle of 65° that enables the detection of the LO_3 _mode of silica at about 1250 cm^-1 ^in addition to the TO_3 _mode located near 1080 cm^-1^.

In SRSO/SiO_2 _around 1225 and 1080 cm^-1 ^we notice the LO_3 _and TO_3 _peak from the Si-O stretching, the TO_4_-LO_4 _doublet between the 1100-1200 cm^-1 ^and the TO_2_-LO_2 _asymmetric stretching of Si-O from SiO_2 _at 810 and 820 cm^-1^, respectively [13]. The presence of Si-nc is attested by the intensity of the LO_3 _peak which is representative of the Si-O bond at the interface [14] between silicon and silica while the TO_3 _vibration mode at about 1080 cm^-1 ^is the signature of the volumic silica.

The SiN_*x*_/SiO_2 _film has a broad peak in the 1250-950 cm^-1 ^region which can be due to the contributions of both LO and TO modes from SiO_2 _and Si-N stretching mode [15-17]. The absorption band located around 860 cm^-1 ^could be attributed to the Si-N asymmetric stretching mode.

In the case of SRSO/SiN_*x *_films, the shoulder around 1190 cm^-1 ^may be due either to N-H bond [16,18] or to a contribution of the LO_3 _mode of Si-O-Si bonds at 180° [13]. Such a result is the signature of the Si nanoparticles formation within either the SiN_*x *_[19] and/or the SRSO sublayer [13]. Between 1050 and 1070 cm^-1 ^lies the LO peak of a-Si_*x*_N_*y*_H_*z *_from Si-N as it has been observed in the SiN_*x*_/SiO_2 _spectrum adding the contribution of the TO Si-O mode.

### PL spectra

**Figure 2:**
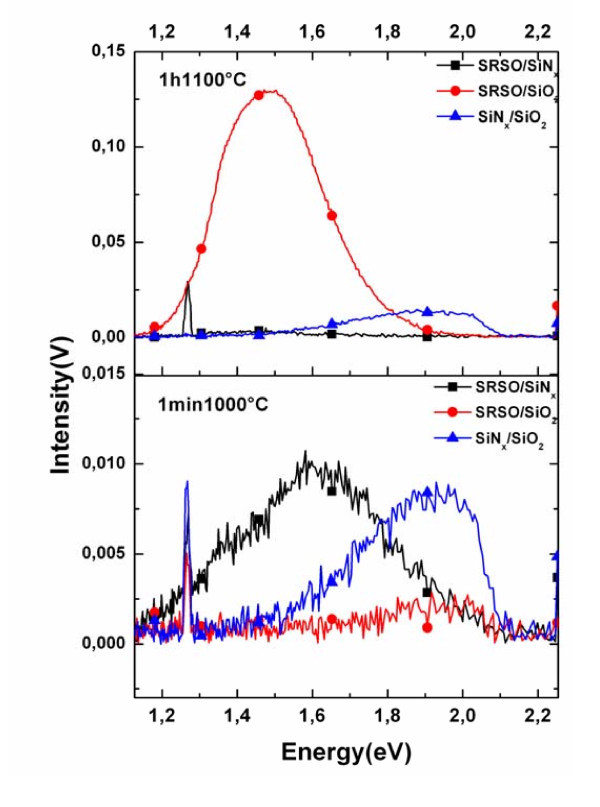
**Effect of annealing treatment on the PL intensity of the multilayer structures**.

The PL emission spectra of the annealed multilayer structures were measured using 488 nm excitation wavelength and the spectrum was recorded in the visible range. Two different annealing treatments were chosen for the study--1 min-1000°C (rapid thermal annealing--RTA) and 1 h-1100°C under N_2 _atmosphere, the latter being the classical annealing treatment used for recovering defects in SiO_2 _matrix to favor luminescence from Si-nc [3]. Figure 2 shows the effect of the annealing treatment on the PL intensity of the three kinds of multilayer structures. All the curves are normalized to a total thickness of 100 nm. Since the number of periods and the sublayer thickness remains the same for each of these films, i.e., *N*_periods_(*t*_sublayer1_/*t*_sublayer2_) = 50(3.5/3.5 nm), it becomes possible to make a comparative analysis from the PL spectrum of these three different multilayer structures. The interference effect in PL intensity has been investigated by the method proposed by Holm et al. [19] for all the spectra presented in this article. This method gives us the PL intensity versus layer and substrate parameters (refractive indices, thicknesses). We assume and homogenous density of emitting centers, an average refractive index within the thickness of multilayer. For measurements on Figure 2 no important change in PL has been found due to interference.

**Figure 3:**
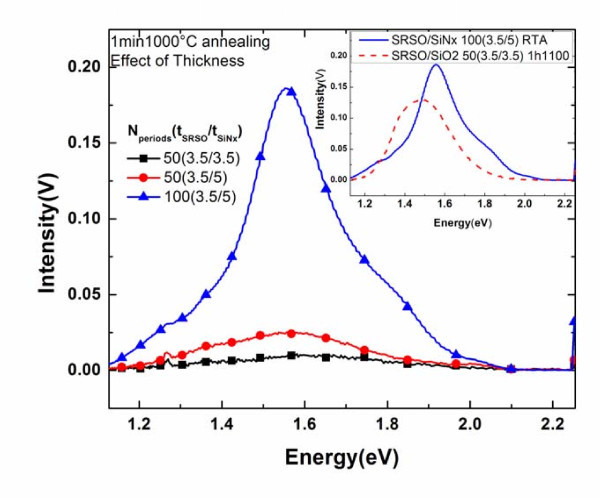
**Effect of sublayer thickness and total thickness of SiN_*x *_on the PL spectrum on RTA**. (Inset: comparison between the SRSO/SiO_2 _annealed at 1 h-1100°C and SRSO/SiN_*x *_structure subjected to RTA).

It can be noticed from the spectrum that when the multilayers are subjected to the classical annealing treatment of 1 h-1100°C, there is no emission from the SRSO/SiN_*x *_while the SRSO/SiO_2 _structure shows a strong PL signal and has a wide range of emission spectrum. At the same time, it is interesting to note a very weak PL signal in the case of SiN_*x*_/SiO_2_. The PL peaks appear in a region usually related to the optical transitions in the SiO_2 _matrix due to the presence of defects [3,17]. The lower part of Figure 2 shows the PL spectrum recorded after annealing the multilayer structures for 1 min at 1000°C (RTA). The response of the multilayers to this annealing treatment shows almost a reversed trend of what was observed in the case of classical annealing treatment. It can be noted that the SRSO/SiN_*x *_has the highest intensity. No PL emission has been recorded from the SRSO/SiO_2 _system. We may note from the figures that the luminescence peak arising from the SiN_*x*_/SiO_2 _structure around 1.9 eV is the same whatever the annealing temperature. The fitting of the PL curve recorded from the SRSO/SiN_*x *_film evidences the presence of two emission bands centered at 1.65 and 1.37 eV. Though this result is interesting and shows the possibility of exploiting SRSO alternated with the SiN_*x *_sublayer to achieve a control over the thermal budget, it also has to be mentioned that the PL intensity obtained is one order of magnitude lower than the emission of SRSO/SiO_2 _subjected to classical annealing. Hence, two methods of fabrication were attempted with the aim of increasing the PL intensity: (i) increasing the SiN_*x *_sublayer thickness to 5 nm and (ii) doubling the number of periods, i.e., fabricating 100 periods of 3.5 nm SRSO alternated with 5 nm SiN_*x*_. Figure 3 shows the effect of the aforesaid fabrication methods on the PL spectrum of the SRSO/SiN_*x *_multilayers. All the spectra have been normalized to 100 nm thickness for comparison. The interference effect in PL intensity has been also investigated by the previously mentioned method PL intensity from both 50 periods multilayers should be decreased by about 15%, in order to take into account the enhancement effect due to maxima of interference. The first method adopted reveals that the SiN_*x *_thickness has some significant contribution toward the luminescence. There is a slight change in the emission wavelength from 1.59 eV with 3.5 nm SiN_*x *_sublayer to 1.55 eV in the case of 5 nm SiN_*x *_sublayer. Irrespective of the number of periods deposited, for a given sublayer thickness the wavelength of emission peak remained constant. It is interesting to note that the emission intensity increases with the SiN_*x *_thickness. This result motivated toward trying out the second method mentioned and it can be noticed that the PL signal increases 7.4 times when the number of (3.5 nm)SRSO/(5 nm)SiN_*x *_pattern is increased from 50 to 100. For that case one can notice is the presence of a small peak between 1.90 and 1.65 eV and another one around 1.5 eV. The inset in Figure 3 shows a comparison between the SRSO/SiO_2 _annealed at 1 h-1100°C and SRSO/SiN_*x *_structure subjected to RTA. One can notice that the emission peak from the SRSO/SiN_*x *_system shifts in the visible region and this is one of the advantageous aspects for the solar cell application. It is very interesting to note that the SRSO/SiN_*x *_annealed for a very short time of 1 min at 1000°C is 1.43 times more intense than the SRSO/SiO_2 _structure annealed for a long time of 1 h and at higher temperature. Accounting for the interference effect, we can infer that SRSO/SiN_*x *_exhibits higher PL intensity than SRSO/SiO_2_. Thus, it can be seen that a replacement of the SiO_2 _sublayer by the SiN_*x *_sublayer and alternating it with the SRSO sublayer not only favors luminescence but paves way to achieve a control over the thermal budget.

## Discussion

The PL spectra of the SRSO/SiN_*x *_subjected to two different annealing treatments show that the quenching of the PL signal after an RTA can be attributed to the non-radiative defects either at the interface of Si-nc and the SiO_2 _matrix or within the SiO_2 _matrix itself which traps the photon arising from the recombination of the exciton within the Si-nc. On the contrary, it can be seen that the SiN_*x *_sublayer favors luminescence even if this later could be attributed to the defects in the matrix. Noticing the shift in emission peak from 1.9 to 1.6 eV in the case of SiN_*x*_/SiO_2 _and SRSO/SiN_*x*_, respectively, it can be said that the sandwiching of SRSO between SiN_*x *_instead of SiO_2 _sublayers not only favors luminescence but also exhibits luminescence in a region attributed to the emission from Si-nc. This implies that though at this temperature SiN_*x *_shows a defect-related PL, when alternated with SRSO, the emission from Si-nc becomes dominant.

On the other hand, the quenching of PL in classically annealed SRSO/SiN_*x *_is quite surprising as several authors have noticed an increase of the PL signal either from SRSO or SiN_*x *_after such annealing. It also should be noted that the 'SRSO sublayer' fabricated under the same conditions and alternated with SiO_2 _sublayer has a high emission. Hence one can conclude that the presence of the SiN_*x *_sublayer quenches the PL. This can be attributed either to the coalescence of Si clusters at such an annealing treatment thereby overcoming the quantum confinement regime or to the non-radiative defects at the interface between SRSO and SiN_*x *_or in SiN_*x*_. The increase of the PL emission when increasing the number of layer could be the result of H diffusion during the deposition process which favors the recovering of the defects and the Si nanoparticles formation. Such a hypothesis is supported by the presence of N-H bonds revealed by FTIR experiments in the non-annealed multilayers and that can be attributed to the Si-nc formation [17]. Another explanation could be the increase of strain with the number of layer that favors the Si-np formation resulting in an increase of the Si-np density and hence in the PL emission. However, the comparison in the inset of Figure 3 of the two types of multilayers demonstrates the advantage to replace the SiO_2 _sublayer by the SiN_*x*_. HRTEM experiments are in progress to understand the optical behavior of these multilayers.

## Conclusion

The multilayers were fabricated using the sputtering technique and the FTIR spectrum revealed its characteristic peaks. Although SiO_2 _is the most sought host matrix, we evidenced the interest of replacing it with the SiN_*x *_matrix. A higher intensity of PL emission was obtained for RTA when SiN_*x *_matrix was used whereas from the SiO_2 _matrix there was no considerable intensity at such an annealing treatment. We have achieved comparable intensity of emission within one minute of annealing and at a lesser temperature, in comparison to the classical annealing treatment that is done for longer time and slightly higher temperature. We also observe an increase in the PL emission with increase in the number of periods. High-resolution electron microscopy experiments are in progress to understand the effect of the annealing process on the achieved optical properties. This set of above-mentioned results paves the way for the fabrication of novel structures for solar cell device applications similar to the one recently reported by Di et al. [20].

## Abbreviations

PL: photoluminescence; RTA: rapid thermal annealing; Si-nc: Si-nanoclusters; SRSO: silicon-rich silicon oxide.

## Competing interests

The authors declare that they have no competing interests.

## Authors' contributions

RPN fabricated the multilayers under investigation and carried out the characterization studies.CD and JC made significant contribution to the optical properties and interference effect. FG conceived of the study and participated in the coordination and writing of the manuscript. All authors read and approved the final manuscript.
